# Horsford’s Acid Phosphate

**Published:** 1873-06

**Authors:** 


### HORSFORD'S ACID PHOSPHATE.

Having used this palatable and efficient remedy with satisfactory results, we can speak advisedly as to its merits. The cereals are so thoroughly deprived of their phosphates by the refining processes to which they are submitted, that the supply of these important elements of nutrition falls far short of the demand, especially by the exhausted brain and nervous systems of those employed in severe mental labor, and in childhood, when the rapid development of tissues requires so large a proportion of these elements.

Physiological observations have shown beyond a doubt that a certain quantity of phosphates are required to supply the first basis for every new tissue, and there is no fibre of the human body that does not contain phosphate of lime. Many diseases, especially disorders of the nervous system, are now attributed to a diminution of this substance. Horsford's Acid Phosphate supplies this want in the highest degree. Send for descriptive pamphlet containing the opinions of the highest medical authorities on its use and value. Address W. H. M. Anthony, 51 Murray street, New York.

*The Obstetrical Journal of Great Britain and Ireland, including Midwifery and the Diseases of Women and Children.* Edited by James H. Aveling, M.D., and Alfred Wiltshire, M.D., with an American supplement edited by William F. Jenks, M.D., Assistant Physician to the Philadelphia Dispensary.

This Journal is made a valuable acquisition to our gynæcological and obstetrical literature. Those interested especially in these branches of medicine can not well afford to be without it, while the general practitioner will find it a gem to his medical collections. Volume 1 No. 1, the copy before us, promises tc give it a front rank in medical journalism. We extend to this new Journal a cordial welcome, and heartily wish it the abundant success it so richly merits.

To be issued monthly, beginning with April 1873. Price $5 per annum; single numbers fifty cents. Henry C. Lea, Philadelphia.

*New Orleans Medical and Surgical Journal.* Edited by S. M. Bemiss, M.D., Professor of the Practice of Medicine in the University of Louisiana.

We have also the announcement of a revival of the *New Orleans Medical and Surgical Journal*, to be edited by S. W. Bemiss, M.D., Professor of the Practice of Medicine in the University of Louisiana, the full success of which will afford us pleasure, earnestly desiring as we do the building up of Southern medical literature as far above any selfish personal interest in journalism.

